# *NeuroRetriever*: Automatic Neuron Segmentation for Connectome Assembly

**DOI:** 10.3389/fnsys.2021.687182

**Published:** 2021-07-23

**Authors:** Chi-Tin Shih, Nan-Yow Chen, Ting-Yuan Wang, Guan-Wei He, Guo-Tzau Wang, Yen-Jen Lin, Ting-Kuo Lee, Ann-Shyn Chiang

**Affiliations:** ^1^Department of Applied Physics, Tunghai University, Taichung, Taiwan; ^2^Brain Research Center, National Tsing Hua University, Hsinchu, Taiwan; ^3^National Center for High-Performance Computing, National Applied Research Laboratories, Hsinchu, Taiwan; ^4^Institute of Biotechnology and Department of Life Science, National Tsing Hua University, Hsinchu, Taiwan; ^5^Department of Computer Science, National Yang Ming Chiao Tung University, Hsinchu, Taiwan; ^6^Institute of Physics, Academia Sinica, Taipei, Taiwan; ^7^Department of Physics, National Sun Yat-sen University, Kaohsiung, Taiwan; ^8^Institute of Systems Neuroscience, National Tsing Hua University, Hsinchu, Taiwan; ^9^Department of Biomedical Science and Environmental Biology, Kaohsiung Medical University, Kaohsiung, Taiwan; ^10^Kavli Institute for Brain and Mind, University of California, San Diego, San Diego, CA, United States

**Keywords:** neuroimage processing, drosophila, segmenation, tracing, connectome

## Abstract

Segmenting individual neurons from a large number of noisy raw images is the first step in building a comprehensive map of neuron-to-neuron connections for predicting information flow in the brain. Thousands of fluorescence-labeled brain neurons have been imaged. However, mapping a complete connectome remains challenging because imaged neurons are often entangled and manual segmentation of a large population of single neurons is laborious and prone to bias. In this study, we report an automatic algorithm, *NeuroRetriever*, for unbiased large-scale segmentation of confocal fluorescence images of single neurons in the adult *Drosophila* brain. *NeuroRetriever* uses a high-dynamic-range thresholding method to segment three-dimensional morphology of single neurons based on branch-specific structural features. Applying *NeuroRetriever* to automatically segment single neurons in 22,037 raw brain images, we successfully retrieved 28,125 individual neurons validated by human segmentation. Thus, automated *NeuroRetriever* will greatly accelerate 3D reconstruction of the single neurons for constructing the complete connectomes.

To understand information flow and its computation in the brain of healthy and diseased states (Alivisatos et al., 2012), we need to have a comprehensive map of all neuron-to-neuron connections. Using electron microscopy, complete connectomes at synaptic resolution have been mapped in the small nematode with 302 neurons (White et al., 1986), the mushroom body of first instar *Drosophila* larval (Eichler et al., 2017), and the whole adult *Drosophila* brain (Zheng et al., 2018; Scheffer et al., 2020). These connectomes provide the most comprehensive connectivity information but does not tell us who the neurons are and what they are doing. To study how brain neurons change their gene expression and neural activity to orchestrate specific behavior, light microscopy imaging of connectome at single neuron resolution remain essential.

By rendering the brain optically transparent with tissue-clearing reagents, confocal and multi-photon microscopy are commonly used to image large populations of single neurons in the brain (Oheim et al., 2001; Ntziachristos, 2010; Chiang et al., 2011; Hama et al., 2011; Chung and Deisseroth, 2013; Erturk et al., 2014; Richardson and Lichtman, 2015; Huang et al., 2021). With the aim of generating every neuron of connectome, scientists have collected a large number of 3D reconstructed neurons (Peng et al., 2010; Shih et al., 2015) (see also http://www.flycircuit.tw/ and https://github.com/bigneuron). Image processing to categorize single neurons for connectome reconstruction involves following steps: (i) preprocessing and denoising the raw image, (ii) segmenting the boundary of a single neuron for 3D morphology reconstruction (Ang et al., 2003), (iii) tracing and skeletonization from segmented volume data to extract structural features and reduce image size, (iv) warping to archive identified neurons into a common framework for rapid searching and analysis, and (v) 3D visualization of the target neurons in the established model brain. Many automatic/semi-automatic tracing algorithms have been proposed to combine pre- and post-processing methods into pipelines for large-scale skeletonization of single neurons (Peng et al., 2010, 2011, 2015, 2017; Halavi et al., 2012; Lee et al., 2012; Xiao and Peng, 2013; Quan et al., 2016; Magliaro et al., 2017, 2019; Wang et al., 2017; Kayasandik et al., 2018). Nevertheless, background denoise depends largely on sample's intrinsic property and quality of image acquisition. Segmenting a single neuron from original fluorescent image is challenging because its cell boundary is often obscure, due to the intensity variation of genetic labeling, the point spread function of fluorescence and limited optical resolution (see below). Thus, far, in all optical images, single-neuron segmentation has been considered the rate-limiting step in connectomics. For large-scale neuron reconstruction from original raw images, manual segmentation is still considered the gold standard.

For connectomics mapping of large number of single neurons, high-throughput and unbiased segmentation is necessary. However, even in the cleared brain with increased signal-to-noise ratio, background noise still varies among images optimized for each sample with different gene expression and genetic background. Further, fluorescence intensities within the same brain are usually uneven between different neurons or at different parts of the same neuron, and background noise is irregular at different depths. Thus, applying a global cut-off intensity faces a dilemma—a low threshold would not filter out the noise effectively, whereas a high threshold may divide actual neural fibers into separate parts if they are connected by voxels with an intensity level lower than the threshold (Agaian et al., 2007; Pool et al., 2008). This is less a problem in manual segmentation because the human eye judges boundaries based mainly on local features and intensity differences. However, human segmentation is subjective, labor-intensive, and time consuming (Peng et al., 2013). Recently, some state-of-art algorithms have been proposed for single neuron reconstruction according to local properties of the images (Quan et al., 2016; Radojevic and Meijering, 2019; Callara et al., 2020).

The *Drosophila* brain has ~135,000 neurons. Imaging random single neurons expressing fluorescent proteins (Lee and Luo, 2001), the *FlyCircuit* image database (version 1.2) has cataloged more than 22,000 single neurons derived from the manual segmentation of thousands of sample brains (Chiang et al., 2011; Shih et al., 2015). Here, inspired by how human eye judges the boundaries of an object, we propose an automated and unbiased segmentation algorithm, *NeuroRetriever*, using local branch features and high dynamic range (HDR) threshold to identify single neurons from the raw confocal images of the *Drosophila* adult brain. Using *NeuroRetriever*, we successfully retrieved more than 28,000 single neurons validated by manual segmentation.

## Results

### How *NeuroRetriever* Works

To accomplish the goal mentioned above, we (1) developed a tracing algorithm named the *Fast Automated Structure Tracing* (*FAST*) algorithm that extracts the details of a single-neuron skeleton with high accuracy and efficiency at multiple intensity thresholds (or level sets); (2) introduced the concept of “branch robustness score” (BRS) based on domain knowledge of neuronal morphology to assess the position of each voxel within the structure; (3) adapted an HDR thresholding mask on the basis of BRS to segment the target neuron; and (4) integrated the algorithms above into an executable package named *NeuroRetriever* (*NR*), which automatically segments and reconstructs a large population of fluorescent single-neuron images for connectome assembly.

**Figure 1:**
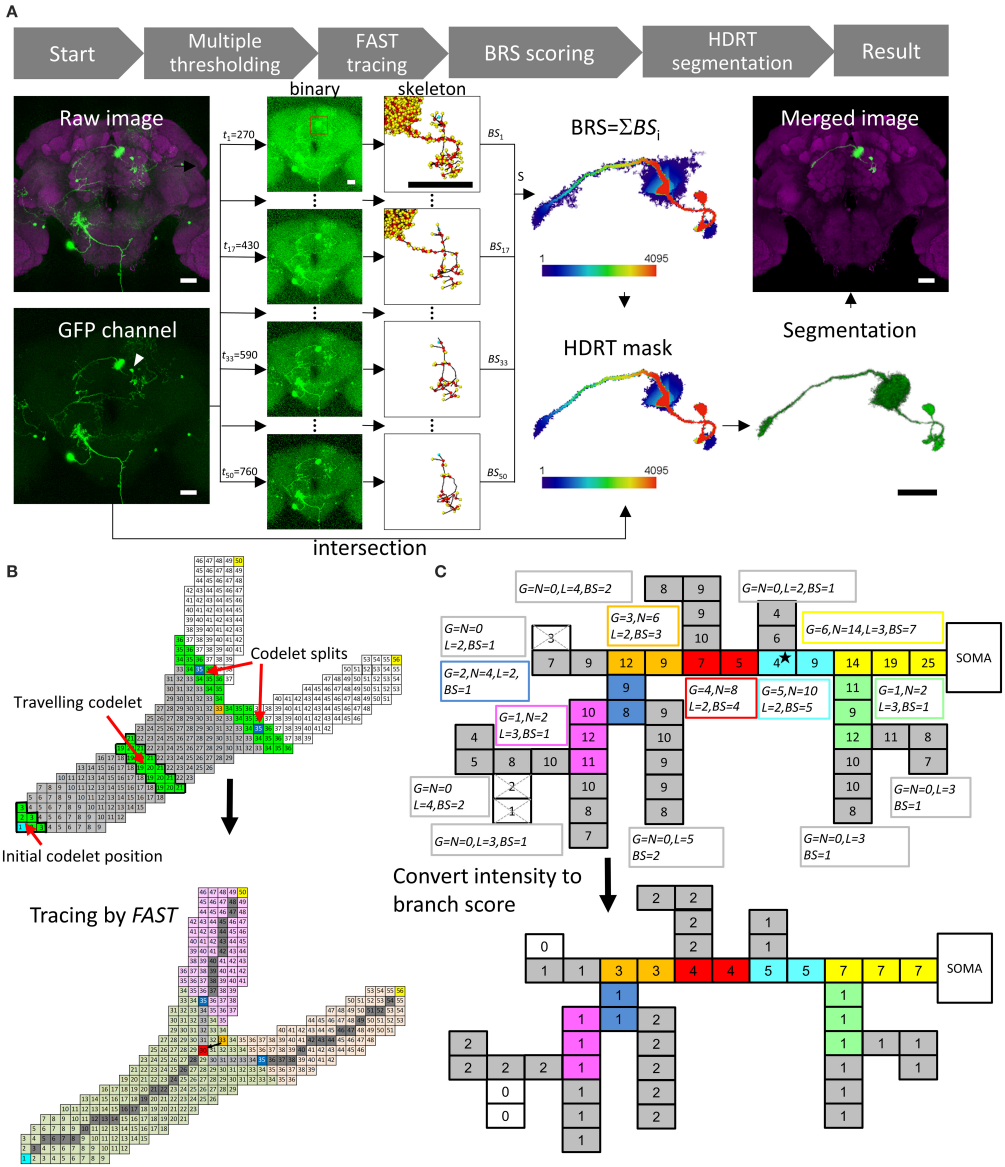
*NeuroRetriever* segmentation procedure. **(A)** *NR* workflow. Left to right: MARCM-labeled neurons (green) in the whole brain (magenta) 🡪50 datasets (*t*_1_-*t*_50_) with serial intensity thresholds from low (top) to high (bottom) 🡪Apply *FAST* to trace skeletal structure for each dataset and calculate BS 🡪Sum 50 BS datasets to obtain BRS for each voxel 🡪Set BRS threshold to generate HDR thresholding mask 🡪Intersect HDR thresholding mask with raw image to segment single-neuron image, and integrate segmented neuron back into original sample brain. **(B)** Schematic of *FAST* algorithm. Upper: Numbered source field for each voxel (square), starting with soma (cyan) as 1. Codelet (green), comprising three rows of linked voxels with consecutive source field values, propagates from soma to fiber terminals. Initial codelet (voxels with source field 1, 2, and 3) travels along the branch, and splits into two at branch point (orange, source field = 33). Each branch has new start point at center (blue). Lower: New branch point (red) determined by retracting two voxels from original codelet branch point (orange). Skeleton determined as serial central points (gray) linking soma (cyan) and branch point (red) to terminals (yellow). **(C)** Upper: Schematic example of BS calculation with *G*_0_ = 2, *L*_0_ = 2 voxels and *N*_0_ = 3*G*_0_ = 6. Numbers in voxels are original fluorescent intensity. Gray branches are terminals. Consecutive voxels with same color belong to same branch. Boxes beside branches demonstrate BS calculation. The super- and subscripts of $G_{i}^{\left(j\right)}$, $N_{i}^{\left(j\right)}$, $L_{i}^{\left(j\right)}$, and $BS_{k}^{\left(j\right)}$ have all been omitted and shortened to *G, N, L*, and *BS*, respectively. Lower: Resulting BS for each voxel. Scale bars represent 25 μm.

Figure 1A shows the workflow of *NR*. Multiple global intensity thresholds, *t*, were applied to the raw data and yielded a series of images with different levels of noise. Next, *FAST* was applied to each image to obtain skeletal information. The next step was to calculate the branch score (BS) for all voxels in each thresholded image. Summing up BS for all thresholds gave a BRS for each voxel. The HDR thresholding mask was then automatically generated using the set of voxels with a BRS larger than a default *m* value (*m* = 40 for the *FlyCircuit* dataset). With the HDR thresholding mask containing a wide range of intensity thresholds, the program then automatically segmented the single neuron by intersecting between the mask and the raw image. Smaller *m* value gives more details of the neuronal morphology. Users can optimize the segmentation result by adjusting the *m* value.

The main engine of this procedure, *FAST*, was designed for tracing tree-like images, such as neurons and blood vessels (Supplementary Figure 1). All voxels in an image were first coded by a value “source field,” which was the path distance from the starting point plus 1 (the numbers in the squares representing the voxels in Figure 1B). Source field of starting point (soma of the neuron) was 1. A “codelet” at position *i* was the set of voxels whose source field values were *i–*1, *i*, and *i* + 1. At the beginning of tracing, the initial codelet position is at (*i* = 2) which means the codelet contains the voxels with source field values 1, 2, and 3. Then the codelet was launched from the cell body (increasing its position *i* by 1 for each step) and traveled through the whole voxel set to trace the structure (green voxels in Figure 1B, upper panel). *FAST* determines the branching points, endpoints, and central points of each branch, which were the points on the trajectory of the center of mass of the codelet (Figure 1B, lower panel).

Figure 1C presents a schematic illustration of the BS/BRS scoring system of a single neuron. In the upper panel, the squares represent voxels and the number in each square represents its green fluorescent protein (GFP) intensity. In this case, a global intensity threshold, *t* = 4, was applied. Note that the GFP intensity in the proximal upstream branches was generally greater than that in distal downstream branches and the intensity of actual signals was usually greater than that of noise. Nevertheless, exceptional weak points (the star in Figure 1C) often exist due to the inevitable fluctuation of intensity in the imaging procedure. Upon applying thresholds greater than the intensity of such weak points, they would be eliminated and downstream branches would be excluded from the segmentation. The lower panel of Figure 1C shows the BS for each voxel at this *t* by measuring the downstream branch generation numbers, downstream branch numbers, and length of each branch. This essential information arose from the *FAST* tracing results. In this example, we see that the BS for the upstream fibers, including the weak point mentioned above, was higher than that for the downstream fibers. Note that, importantly, the BS still maintains intensity information because voxels with higher intensities survived under higher thresholds and were counted more times than low-intensity voxels. Accumulating the BS from the whole range of *t*, we obtained the final BRS for each voxel in the image. Thus, the global structure of the whole segment and intensity of individual voxels were both taken into account in the BRS.

### Evaluation of the HDR Thresholding

**Figure 2:**
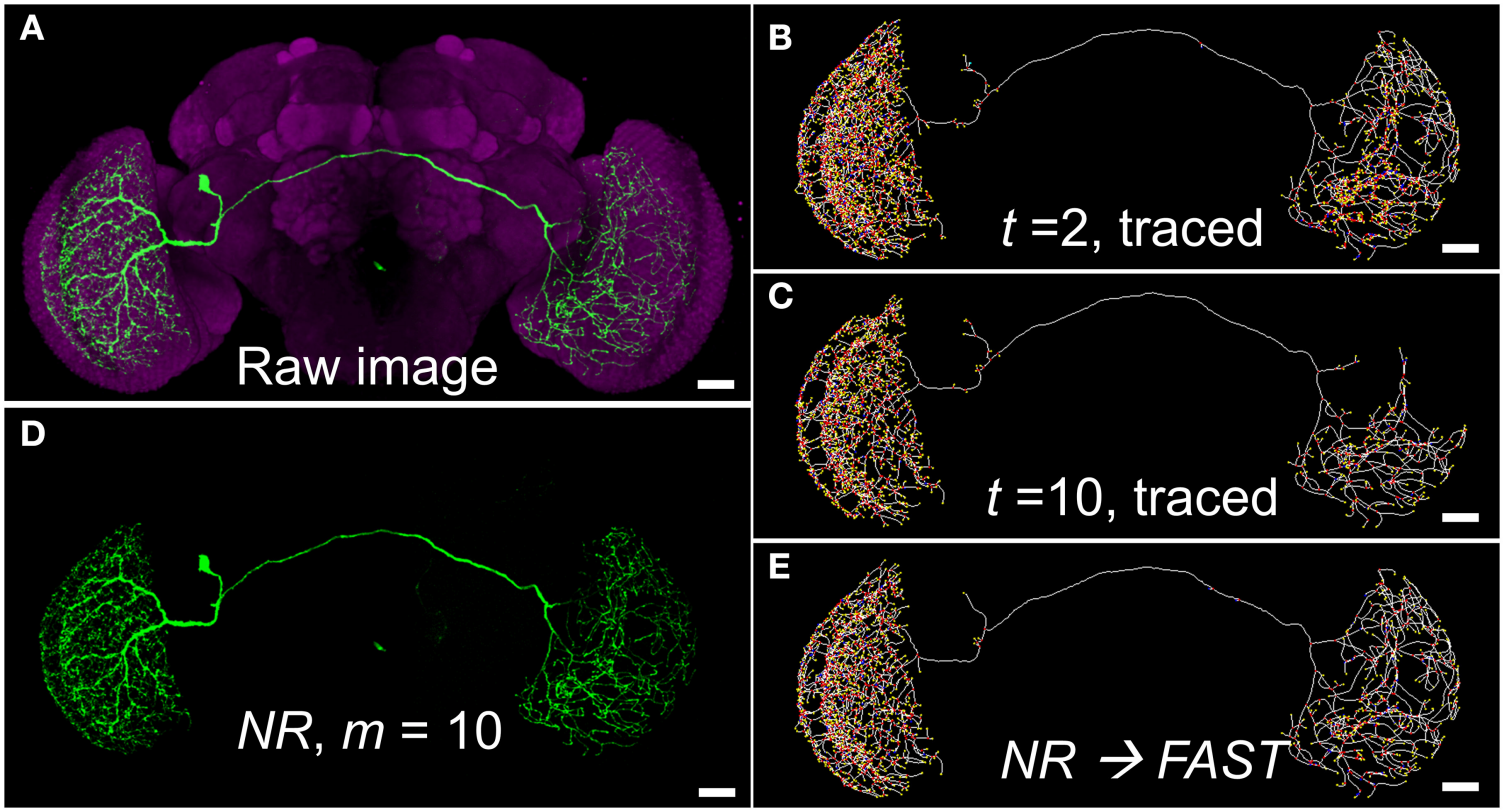
An example of segmentation dilemma solved by *NR*. **(A)** The raw image of a visual neuron (*FlyCircuit* Neuron ID: Trh-F-000025, 8-bit, intensity 0–255). **(B,C)** Skeletons traced by *FAST* with global thresholds *t* = 2 and 10, respectively. **(D)** The neuron segmented by *NR* with HDR thresholding mask at *m* =10, fine-tuned from the default value of *m* = 40, for the best segmentation. **(E)** Skeleton of **(D)** traced by *FAST*. Scale bars represent 25 μm.

Figure 2 shows an example of differences between using the HDR thresholding and the uniform thresholding for segmenting a single neuron with variable label intensity and background noise at different depth. This neuron innervated both sides of the optic lobes and had an extremely complex arborization. The image was the maximal projection of serial raw images constructed by stitching two stacks of optical sections through the left and right brain hemispheres, respectively, because of its large size (Figure 2A). Therefore, the background noise levels for the left and right parts were intrinsically different, providing an ideal example for the test (Supplementary Figure 2). There were many key voxels with a very low intensity in the right part, which could make the structure fragile, even though the intensity threshold was not very high. If we used a lower threshold on the raw image, the detailed structure of the right side would survive but that of the left side would be very noisy (Figure 2B, Supplementary Figures 2A–C). On the other hand, a higher-intensity threshold filtered out more noise and made the left part clearer, but the structure of the right part was severely disrupted (Figure 2C, Supplementary Figures 2D–J). The *NR* segmentation using a wide range of intensity thresholds solved the dilemma of effectively denoise the background while keeping fine structures with weak signal at different locations. Using the BRS-based HDR thresholding, *NR* acts like the human eye with dynamic detection of intensity differences in local boundaries along the fibers of a single neuron for 3D image segmentation (Figure 2D). Once the neuron was segmented, we then used the *FAST* algorithm to trace the skeleton again for efficient connectomic analysis (Figure 2E). In this case, the structure of the right part was well-preserved, while the left part also became much clearer.

### *NeuroRetriever* vs. Human Segmentation

Automatic *NR* segmentation retrieved 28,125 single neurons from 22,037 raw brain images archived in the *FlyCircuit* database. To evaluate the effectiveness and quality of *NR* segmentation, all neurons were also segmented by experienced operators using the same raw images (called “human segmentation” in the following) and served as the “gold standard” for the segmentation.

**Figure 3:**
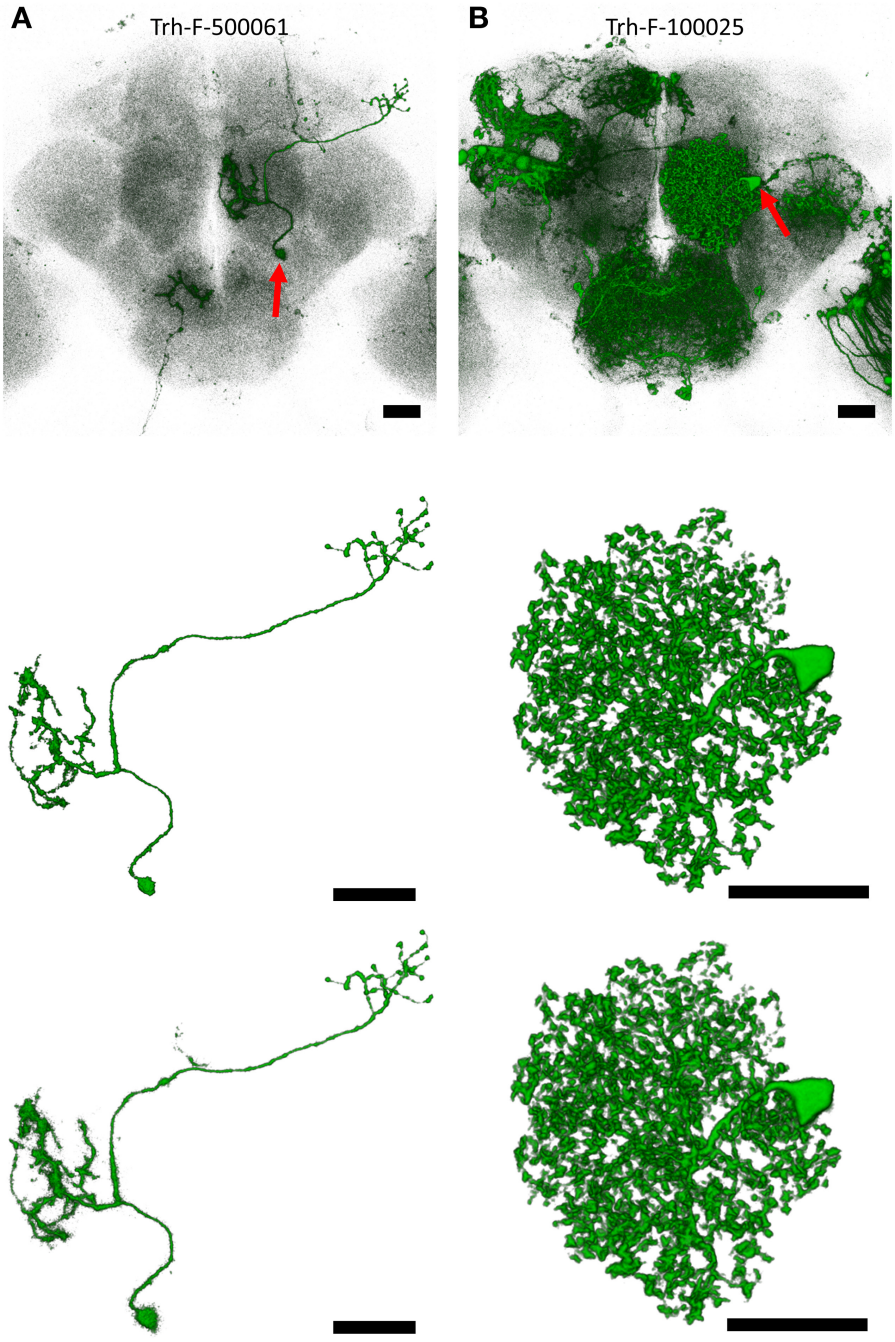
Examples of single neurons segmented by *NR* vs. human. **(A)** An olfactory projection neuron (Trh-F-500061) linking the antennal lobe and lateral horn. **(B)** A local interneuron (Trh-F-100025) within the antennal lobe. Upper row: raw images, middle row: human-segmented neurons, lower row: *NR*-segmented neurons. Red arrows indicate soma. Scale bars represent 25 μm.

Figures 3A,B show two raw images of single neuron in a noisy background representing different types of challenges for the qualitative assessment of *NR*-segmented results compared with their human-segmented counterparts. The first example was a projection neuron sending long fibers innervating several brain regions at different depths (Figure 3A). Another example is a local interneuron with numerous short fibers close to each other within a small region (Figure 3B). In both cases, the automatic *NR* segmentation retrieved additional fine details but also more noises resulting in blurrier image than human segmentation. Upon closer observation, we found that at least some of these *NR*-segmented blurry fine structures are weakly labeled tiny branches and small protrusions along the fiber. Importantly, *NR* and human segmented neurons exhibited similar morphometric features and *FAST*-traced skeleton for connectomic analysis (see below).

**Figure 4:**
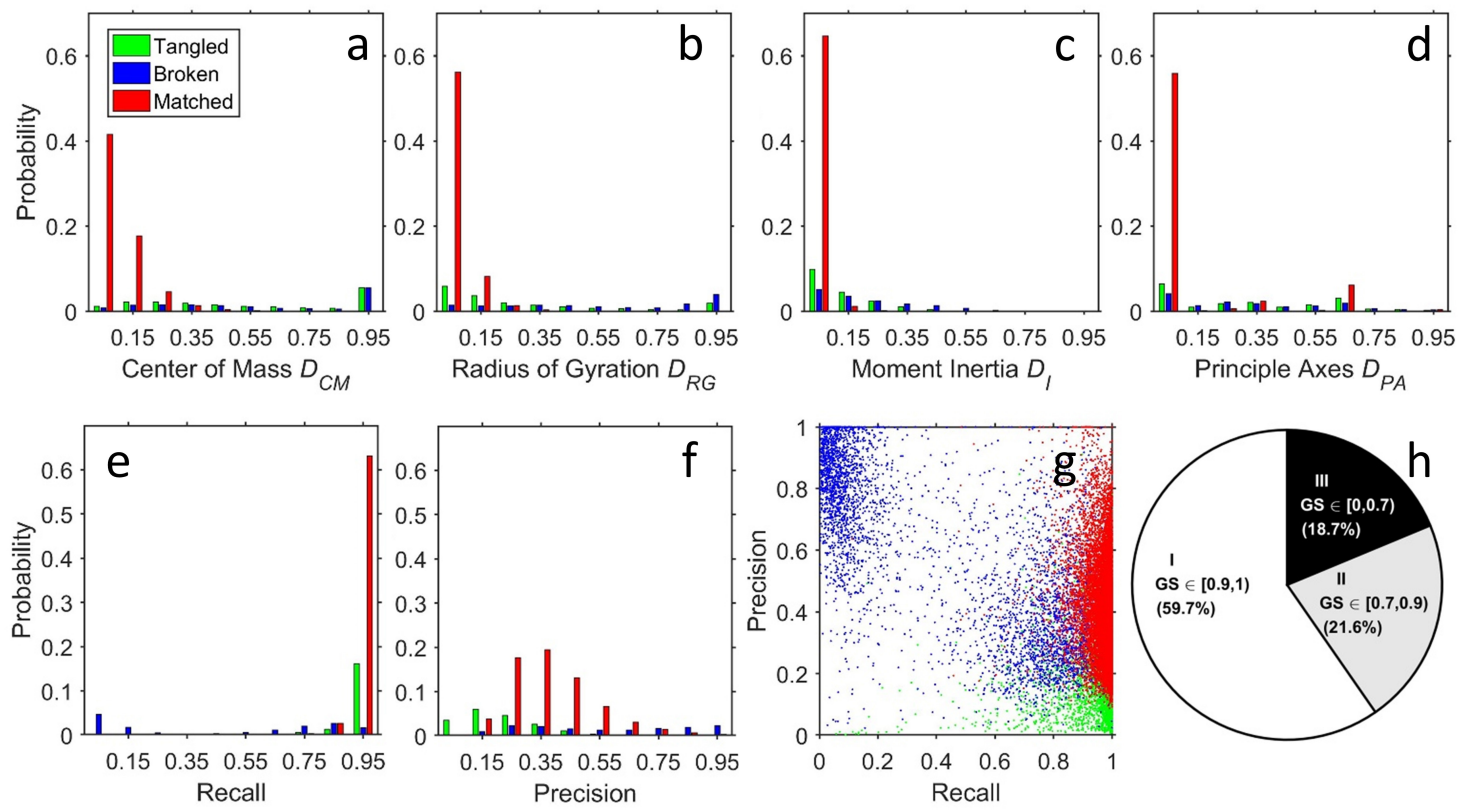
Structural and voxel-to-voxel comparison between *NR* and human segmentation. Compared with human segmentation results, assessment of *NR* results is classified into three types: matched (M, red), broken (B, blue), and tangled (T, green) by visual validation of experts. The distributions of quantitative analyses were **(a)** the distance between centers of mass (position), **(b)** difference in the radii of gyration (size), **(c)** difference in moments of inertia (shape), **(d)** difference in orientation of the three principle axes of rotation (orientation), **(e)** recall, and **(f)** precision. **(g)** Scatter plot for the (recall, precision) for each neuron. The point (recall, precision) = (1, 1) implies a perfect match (identical *NR-* and human- segmentations). As expected, type M neurons (red points) had a recall since they successfully reproduced human segmentation. They also had an intermediate precision because of the greater thickness of the fibers and the preserved weaker fibers, as discussed in Figures 3A,B. The precision could be smaller than 0.5 due to the large surface-volume ratio of the fractal-like neuronal morphology. The tangled type T (green points) has high recall because the voxels in the *NR* result contained the whole target and low precision small because the neuron mixed with other neuron(s). Finally, the points for the broken type B form two groups. The first group is close to the group of red points (matched) but with lower recall, corresponding to the cases that only a few branches are missing. Another group is located at the upper left corner (low recall and high precision), corresponding to the cases that *NR* gets only the part close to the soma and misses most of the branches. **(h)** Pie chart for the ratios of the three classes according to the ranges of the GS.

For quantitative comparison, we measured the distance of the centers of mass, radius of gyration, relative moment of inertia, and directions of principal axes for each segmented neuron, which quantitatively characterized the position, size, shape, orientation of the neuron, respectively (Figures 4a–d). Combining these parameters and a voxel-to-voxel comparison (Figures 4e–g), we then generated a global similarity metric (*GS*) ranging from 0 to 1 (*GS* = 1 for two identical images, see Methods) to evaluate the quality of results generated by *NR* and human segmentation. Among 28,125 segmented neurons, we found 59.7% within *GS* ∈ [0.9, 1)(Class I), 21.6% within *GS* ∈ [0.7, 0.9)(Class II), and 18.7% within *GS* ∈ [0, 0.7)(Class III), respectively (Figure 4h).

Next, we visually inspected all results and classified the differences between *NR* and human segmented neurons into three groups: matched, broken, and tangled (Figures 4a–g). Here, we applied a very strict standard for “matched” segmentation—they could be archived into the single neuron image database directly without any human correction. Overall, the morphology of 65.8% of *NR* segmented neurons matched with human segmentation, which had minor differences within the range as those segmented by different operators using the same raw image. The remaining 34.2% of *NR* segmented neurons contain two kinds of mismatch: broken and tangled. Most broken neurons resulted from discontinuous labeling of fibers in the raw images. And when two or more neurons were tangled in the raw image, *NR* inevitably segmented additional fibers and/or soma.

Comparing the quantitative *GS* with the expert visual validation, we found that almost all matched cases has *GS* > 0.7 (Supplementary Figure 4). For example, *GS* for the neurons in Figures 3A,B are 0.788 and 0.972, respectively. Supplementary Figure 5 shows two rare cases with *GS* > 0.95 but classified as broken and tangled under the strict condition of visual validation. In such cases, they need to be human corrected before archived into the database. Overall, ~65% of matched neurons could be directly deposited to the image database, while the remaining 35% still served as good references and greatly accelerated human segmentation process with only minor intervention. In addition to high degree of similarity, *NR* segmentation also retrieves additional new neurons that are previously overlooked by human segmentation from the same brain samples (e.g., shown in Supplementary Figure 6).

### Comparing the *FAST*-Traced Skeletons

**Figure 5:**
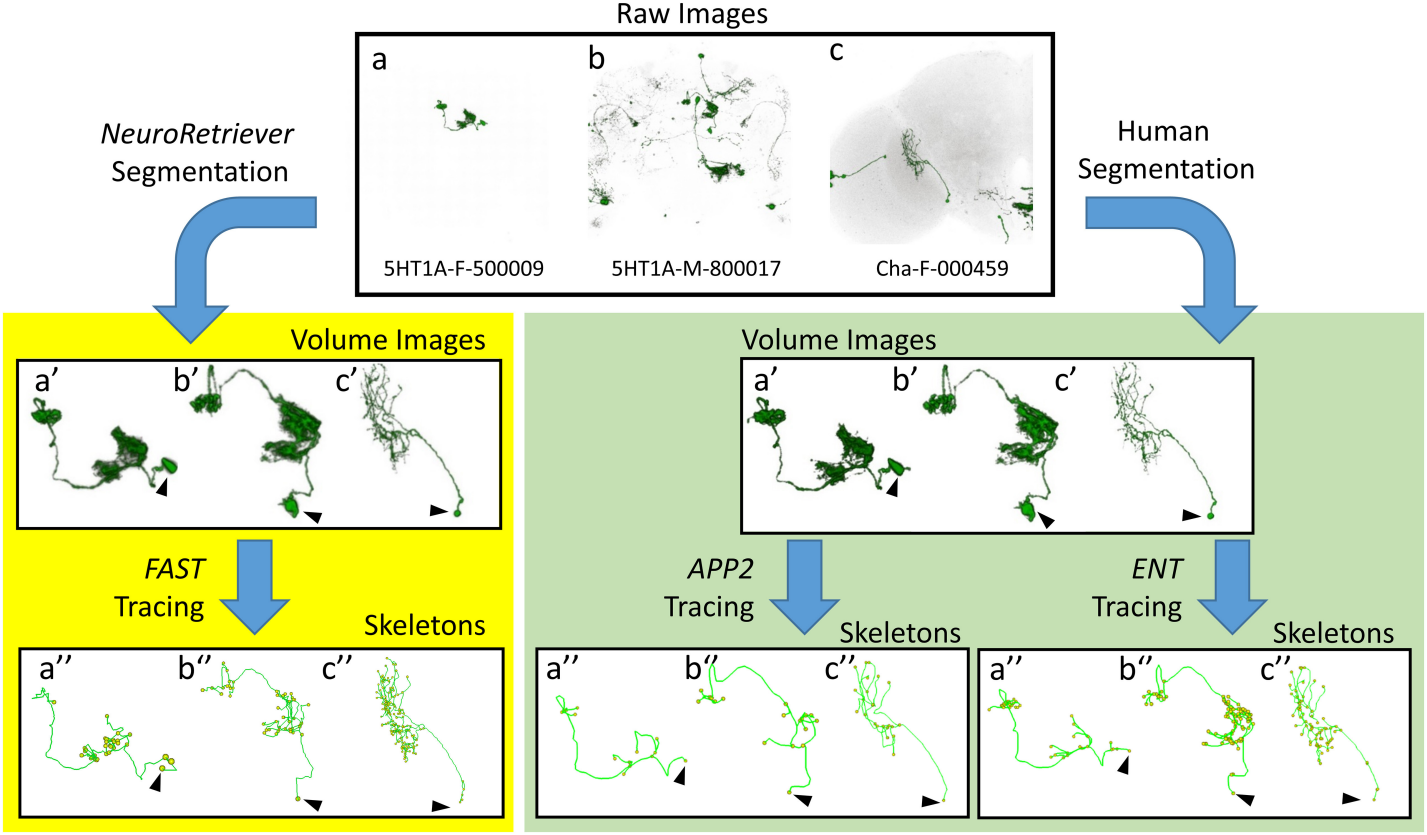
Comparison between *FAST*-traced skeleton and *APP2-* and *ENT*-traced skeletons. Upper panel: Examples of three different types of raw image quality. (a) 5HT1A-F-500009: a single neuron in the brain with clean background. (b) 5HT1A-M-800017: multiple single neurons with clean background. (c) Cha-F-000459: multiple neurons with noisy background. Yellow panel: *FAST*-traced skeleton from the *NR*-segmented neurons. Green panel: *APP2*- and *ENT*-traced skeleton from the human-segmented neurons. Arrowhead indicates the cell body.

Next, we compared *FAST* with other popular algorithms, i.e. *APP2* (Xiao and Peng, 2013) and *ENT* (Wang et al., 2017), for tracing and skeletonization of single neurons from three different types of raw images: single neuron with clean background, multiple neurons with clean background, and multiple neurons with noisy background (Figure 5). *FAST* successfully generated skeletons from all 28,125 *NR*-segmented single neurons. These *FAST*-traced skeletons consistently represented the morphology of *NR*-segmented neurons similar to the *APP2*- and *ENT*-traced skeletons for the human-segmented neurons. Note that however, without segmentation, all the above three algorithms failed to directly generate reliable skeleton from raw brain images with noisy background (Supplementary Figure 7). The advantage of *NR/FAST* is that it can process images with a wide range of quality generated from large-scale imaging tasks such as the MARCM procedure used in *FlyCircuit*, and get the correct skeleton structure as other algorithms.

## Discussion

Recent advances in automated microscopy have generated a large neuroimage dataset for connectome analysis (Oh et al., 2014; Markram et al., 2015; Costa et al., 2016). Labor-intensive human segmentation is still the major bottleneck for high-throughput analysis of connectomic data (Arganda-Carreras et al., 2015). In this study, we report an automatic algorithm, *NeuroRetriever*, using anatomic features and HDR thresholding to segment single neurons directly from the raw fluorescent images with variable background noises. *NR* segmentation has several advantages over the human segmentation. First, *NR* is a deterministic and non-biased method. Unlike inconsistency between operators in human segmentation, *NR* always produces the same result from the same raw data. Second, in general, *NR*-segmented neurons have more details (Figure 3). During our visual inspection, we often found that some *NR* results were evidently “better” than the human results because human segmentation often skipped minute details and humans occasionally made mistakes (Supplementary Figure 3C). Third, although the computing time for segmenting a single neuron was similar between *NR-* and human-segmentation (ca. 20 min), human operators require rest and *NR* could run 24 hours a day with multiple computers in parallel. Using 200 cores of an AMD Opteron 6174 cluster, we used *NR* to segment all 28,125 single neurons from the whole *FlyCircuit* dataset in <2 weeks. Compared with human segmentation (Chin et al., 2014), *NR* does not require expert knowledge, is applicable to a large population of diverse neuron types, and consistent between different runs for the same data. Nevertheless, since *GS* analysis requires a posteriori test and no human segmentation results would be available during its actual application, we recommend that *NR* users should still visually confirm all results to avoid unexpected errors.

Several automated/semi-automated algorithms for tracing and/or segmentation of individual neurons have had some success for certain types of data (Santamaria-Pang et al., 2015; Zhou et al., 2015; Quan et al., 2016; Hernandez et al., 2018; Callara et al., 2020). For the non-uniform background noise problem, the “smart region-growing algorithm” (SmRG) (Callara et al., 2020) segments the neurons using local thresholding based on the distributions of foreground and background signals of optical microscopy. For large-scale neuron segmentation from the images with dense neurites, the “NeuroGPS-Tree” algorithm (Quan et al., 2016) can detangle the densely packed neurites form the statistical morphological information of neurons, to obtain single neuron images. With HDR local thresholding based on the voxel weighting of the tree-like structure, *NR* can deal with both non-uniform background and large-scale segmentation.

Successful *NR* segmentation depends largely on image quality and resolution. We expect that broken mismatch of *NR* segmented neurons will be greatly reduced in clarified tissue with strong and continuous fluorescent labeling of neuronal fibers. However, solving the entangled mismatch and segmenting from dense fibers require nanoscale resolution. We expect that *NR* can be applied to process fluorescence images taken by super-resolution microscopy and expansion microscopy with resolution beyond the optical limit (Small and Stahlheber, 2014; Sigal et al., 2015). Also, the concept of an HDR thresholding mask is likely applicable for identifying other tree-like structures, such as tracheoles and blood vessels (Lin et al., 2017), or other types of non-fluorescent images, such as X-ray images (Ng et al., 2016). With automated *NR* for high-throughput single-neuron segmentation, connectome mapping for large brains with billions of neurons is now conceivable.

## Methods

### Source of Images

The images used in this study were obtained from the *FlyCircuit* database (Chiang et al., 2011; Peng et al., 2015), version 1.2. The full dataset contains 22,037 fluorescent three-dimensional raw image stacks and 28,573 single-neuron images manually segmented by experienced operators. Raw images were two-channel.lsm files, the ZEISS LSM standard format. The neuronal image in the green fluorescent protein (GFP) channel has far fewer non-zero points in comparison with the disc-large (Dlg) channel. We used a script running on Avizo 9.2 to split the channels into two Avizo mesh (.am) files and automatically selected the GFP channel by file size. The.am files from the GFP channel were stored in an ASCII format that could be directly accessed by *NR*.

### Basic Concept

The central concept of *NR* is to assign a score to each voxel with non-zero intensity according to its “importance in the global neuronal morphology” under a wide range of intensity thresholds. In contrast to traditional denoising and segmentation methods, which treat the importance of a voxel as intuitively equivalent to its own fluorescent intensity or the local intensity distribution around the voxel, *NR* evaluated the possibility of the voxels being a real signal from both the intensity and global structure of the neuroimages. Under typical imaging conditions, noise appears randomly and clusters of neighboring noise do not preferentially adopt any particular shape. On the other hand, the basic feature of neuronal morphology is a tree-like structure composed of quasi-one-dimensional fibers. A set of connected voxels having a large tree-like structure composed of many fibers and branching levels was very unlikely to be random noise. Voxels in such a structure should have higher survival chance or, equivalently, smaller local threshold during the denoising procedure. A similar argument could be applied to the connected voxels that form a very long fiber.

Another major feature of *NR* is the reorganized workflow shown in Figure 1A. With the raw fluorescent image, the first task was to detect the soma position(s) automatically in the image based on the shape of the largest ellipsoid-like clusters of voxels (He et al., 2017). The second step involved applying *FAST* to trace the images under a series of global intensity thresholds, *t*_min_ = *t*_1_, *t*_2_, ⋯ , *t*_*n*_ = *t*_max_. The range and step of *t*_*j*_ are determined by the features of the raw image. In this study, both 8-bit and 12-bit fluorescent images were processed, whose voxel intensities were in the ranges of 0–255 and 0–4095, respectively. The increment, *t*_*step*_ = *t*_*j*+1_ − *t*_*j*_, was set at 2 and 10 for the 8- and 12-bit images, respectively. The *t*_*step*_ value can be smaller, but it will need larger *n* to cover the threshold range, which means more computing time. For large *t*_*step*_, it is possible to drop a lot of structure of the neuron and get a broken segmentation. We suggest users do some tests to determine the best value of *t*_*step*_ and other parameters. The default minimal global intensity threshold, *t*_1_ was *t*_*step*_. For images with very high background noise, almost all voxels were connected into a big volume under the small intensity threshold values. For such high-background low-threshold cases, *FAST* will give a huge number of branches, which were meaningless and time-consuming. For such situation, *NR* would adjust the *t*_1_ value such that the number of traced branches was closest to, but no more than, the upper limit of the branch number, *B*_*max*_ = 10,000. Fifty threshold values were taken for each image (*n* = 50), which meant that the widths of the range of intensity thresholding *R*_*th*_ = *t*_max_-*t*_min_ for the 8- and 12-bit images were 98 and 490, respectively. The parameters *B*_*max*_, *t*_*step*_, and *R*_*th*_ can be chosen by the user according to their requirements and the properties of the raw images.

### *FAST*: Extracting the Structural Features

*FAST* is a powerful tracing algorithm to extract structural features from volume data. The flowchart for *FAST* is shown in Supplementary Figure 1. As a schematic example shown in Figure 1B, the “source field” of the voxels (numbers in the squares) in the image was encoded according to the shortest path lengths from the starting point, namely, the soma of the neuron. A “codelet” was launched from the soma and traveled in the direction of increasing source field. Voxels with a source field between *i* − 1 and *i* + 1 belonged to the *i*th position of the codelet. For example, the initial (*i* = 2) and the (*i* = 20) positions of the codelet are marked by the green voxels with a thick black frame in the upper panel. The codelet traveled through the connected voxels by increasing *i*. At (*i* = 35), the codelet split into two codelets (green) and started to trace the two new branches individually from the two new starting points (respective centers of mass of the two new codelets, blue). The codelets stopped at the next branching points or endpoints (yellow) of the neuron.

The trajectory of the center of mass of the codelets defined the central points of the branch (dark gray points in the lower panel of Figure 1B). The central point at the position where the codelet split defined the branch point (orange point). A “local tracing” procedure was performed to (1) move the branching point back from the edge of the branch to an interior point on the central line (red point) and (2) to fill the gap between the new branching point and the starting points of the two downstream branches with additional central points (light gray points in the lower panel). The final *FAST* results in the lower panel show the partition of branches (light green, pink, and light orange), starting point of each branch (blue), branching point (red), endpoints (yellow), and skeleton (gray) of the neuron.

### BRS: Scoring the Structural Importance of Voxels

*FAST* provided the positions of all key points in the skeleton of each neuron (including branching, central, and end points) and the hierarchical information for each branch of the traced neuron at all thresholds, *t*_*j*_. Figure 1C provides an example of the BRS calculation. The intensity threshold in the example was 4 and the three white voxels were deleted. The voxel set under this threshold was traced and had 15 branches. Here are definitions of the measurements for each branches in a neuron traced by *FAST*:

$G_{i}^{\left(j\right)}$: the number of descendant generations of the *i*th branch at threshold, *t*_*j*_. $G_{i}^{\left(j\right)}=0$ for all terminal branches (the eight gray branches in Figure 1C). If the *i*th branch is not a terminal, it would have the set of child branches *C*(*i*), $G_{i}^{\left(j\right)}=\max\left(\overset{\left(j\right)}{\underset{k\in C\left(i\right)}{G}}\right)+1$. For the example in Figure 1C, we focus on the *i* = primary neurite (yellow) and intensity threshold *j* = 4 as a demonstration. The primary neurite has two child branches whose $G_{i=primary}^{\left(j=4\right)}$ are 1 (green) and 5 (cyan). Therefore, $G_{i=primary}^{\left(j=4\right)}$ of the primary neurite is max(1, 5) + 1 = 6.

$L_{i}^{\left(j\right)}$: the length of the *i*th branch at threshold *t*_*j*_. For the primary neurite in Figure 1C, $L_{i=primary}^{\left(j=4\right)}=3$ voxels.

$N_{i}^{\left(j\right)}$: the number of descendant branches of the *i*th branch at threshold *t*_*j*_. For the primary neurite in Figure 1C, $N_{i=primary}^{\left(j=4\right)}=14$.

At threshold, *t*_*j*_, voxels in a particular branch *i* can obtain BS if the branch has $G_{i}^{\left(j\right)}>G_{0}^{\left(j\right)}$, $N_{i}^{\left(j\right)}>N_{0}^{\left(j\right)}$, and *L*_*i*_ > *L*_0_. For the images in *FlyCircuit*, *L*_0_ = 20 voxels = 6.4 μ*m* (20 times the side length of a voxel in the *x-y* plane) for the whole range of intensity threshold *t*. And the definition of the other two parameters:

$G_{0}^{\left(j\right)}$: For *j* = 1 (minimal intensity threshold), calculate $G_{i}^{\left(1\right)}$ for all branches. $G_{0}^{\left(1\right)}$ is set as the value of 75th percentile of $G_{i}^{\left(1\right)}$ values for all *i*. If this value is smaller than 20, $G_{0}^{\left(1\right)}$ is set to be 20. As the threshold *t*_*j*_ increases, $G_{0}^{\left(j\right)}$ decreases accordingly because more branches will be eliminated at higher thresholds and all $G_{i}^{\left(j\right)}$ will be decreased. In the present study, we use:

(1) $$G_{0}^{\left(j\right)}=\max\left(\left\lceil G_{0}^{\left(1\right)}\times \left(1-\frac{t_{j}}{t_{\max}}\right)\right\rceil ,1\right)$$

where ⌊⌋ and ⌈⌉ are the floor and ceiling functions, respectively.

$N_{0}^{\left(j\right)}$:

(2) $$\begin{array}{l} N_{0}^{\left(j\right)}=3G_{0}^{\left(j\right)} \end{array}$$

According to this principle, $BS_{k}^{\left(j\right)}$, the “branch score” earned by voxel *k* belonging to the *i*(*k*)^th^ branch at the threshold, *t*_*j*_, is defined as:

(3) $$\begin{array}{l} BS_{k}^{\left(j\right)}=\max\left(\left[G_{i(k)}^{\left(j\right)}-G_{0}^{\left(j\right)}\right],0\right)+\left\lfloor \frac{N_{i(k)}^{\left(j\right)}}{N_{0}^{\left(j\right)}}\right\rfloor \\ \;+\left\lfloor \frac{L_{i(k)}^{\left(j\right)}}{L_{0}^{\left(j\right)}}\right\rfloor +\lambda_{i(k)}^{\left(j\right)} \end{array}$$

where

(4) $$\begin{array}{l} \lambda_{i(k)}^{\left(j\right)}=\max(\left\lfloor \frac{L_{p}^{\left(j\right)}}{L_{0}^{\left(j\right)}}\right\rfloor ,\text{ for }p\in \Lambda_{i(k)}^{\left(j\right)},\;G_{i(k)}^{\left(j\right)}<G_{0}^{\left(j\right)}\\ \text{and }N_{i(k)}^{\left(j\right)}<N_{0}^{\left(j\right)}) \end{array}$$

which was the score obtained from the length of the longest offspring branch of the *i*th branch, where $\Lambda_{i}^{\left(j\right)}$ was a set formed by all offspring of the *i*th branch. In the example in Figure 1C, $G_{0}^{\left(j\right)}=2,L_{0}^{\left(j\right)}=2$ voxels, and $N_{0}^{\left(j\right)}=6$. Thus, the *BS* of all voxels in the primary neurite equaled $\left(6-2\right)+\left\lfloor \frac{14}{6}\right\rfloor +\left\lfloor \frac{3}{2}\right\rfloor +0=7$ at an intensity threshold of 4.

The BRS of voxel *k* is evaluated by summing $BS_{k}^{\left(j\right)}$ for all thresholds, *t*_*j*_:

(5) $$\begin{array}{l} BRS\left(k\right)=\underset{j}{\sum }BS_{k}^{\left(j\right)} \end{array}$$

The BRS effectively represents the importance of each voxel in the global neuronal morphology extracted from a wide range of intensity thresholds; the original fluorescent intensity of the voxel was also taken into account because voxels with higher intensity would survive for a wider range of thresholds and thus could be counted more times. The next step was to set a cut-off for the BRS, *m*, to determine the HDR thresholding mask of the image. Voxels with a BRS less than *m* are viewed as noise and discarded from the mask of the image. The set of voxels for the single target neuron is segmented out by intersecting the mask and raw image. Finally, *FAST* was used to trace the segmented voxel set again to extract all structural features and the neuron was digitally reconstructed.

### Quantitative Validation of the *NR* Results

A series of quantities were computed for comparing *NR* and human segmentation results, including the segmented voxel sets and global structural features as follows:

*****D*****_*****CM*****_: normalized centers of mass distance which is the difference of the positions of the two segmentation. ${\vec{R}}_{H}$ and ${\vec{R}}_{NR}$ are centers of mass vectors of human- and *NR*-segmented images, respectively. The voxels with non-zero intensity were treated equally with mass = 1.

(6) $$\begin{array}{l} D_{CM}\equiv \min\left(1,\frac{\left|{\vec{R}}_{NR}-{\vec{R}}_{H}\right|}{r_{H}}\right) \end{array}$$

where *r*_*H*_ is the radius of gyration of human segmentation image. For some heavily tangled cases, $\frac{\left|{\vec{R}}_{NR}-{\vec{R}}_{H}\right|}{r_{H}}$ was larger than 1. We used the “min” operator to keep *D*_*CM*_ between 0 and 1.

*****D*****_*****RG*****_: normalized radius of gyration difference which is the difference of the sizes of the two segmentations.

(7) $$\begin{array}{l} D_{RG}\equiv \min\left(1,\frac{\left|r_{NR}-r_{H}\right|}{r_{H}}\right) \end{array}$$

where *r*_*NR*_ is the radius of gyration of *NR*-segmented images, respectively. Again, for those cases $\frac{\left|r_{NR}-r_{H}\right|}{r_{H}}$ larger than 1, we used the “min” operator to keep *D*_*RG*_ between 0 and 1.

*****D*****_*****I*****_: normalized moment of inertia difference which is the difference of the rough shapes of the two segmentations. For an image, the principal moments of inertia were *I*_1_, *I*_2_, and *I*_3_, with *I*_1_ ≥ *I*_2_ ≥ *I*_3_. The normalized principal moments of inertia vector $\vec{i}$ were then defined as $\vec{i}=\left(1,\frac{I_{2}}{I_{1}},\frac{I_{3}}{I_{1}}\right)$. ${\vec{i}}_{H}$ and ${\vec{i}}_{NR}$ were moments of inertia vectors of human- and *NR*-segmented images, respectively.

(8) $$\begin{array}{l} D_{I}\equiv \min\left(1,|{\vec{i}}_{H}-{\vec{i}}_{NR}|\right) \end{array}$$

*****D*****_*****PA*****_: difference of the orientations of the principal axes which is the difference of the orientations of the two segmentations. For a given image, ${\vec{A}}_{i}$ was the principal axis corresponding to the principal moment of inertia, *I*_*i*_ (*i* = 1, 2, 3).

(9) $$\begin{array}{l} D_{PA}=1-\frac{\sum_{i=1}^{3}|{\vec{A}}_{H,i}\cdot {\vec{A}}_{NR,i}|}{3} \end{array}$$

**Recall**: defined as the number of true positive voxels that existed in the human-segmented image and were correctly detected by *NR*, divided by the number of voxels in the human-segmented image. *V*_*H*_ and *V*_*NR*_ represent the set of the voxels in the human- and *NR-* segmented images, respectively.

(10) $$\begin{array}{l} R=\frac{\left|V_{NR}\cap V_{H}\right|}{\left|V_{H}\right|} \end{array}$$

**Precision**: defined as the number of voxels in the intersection of the human- and *NR-*segmented image divided by the number of voxels in the *NR*-segmented image.

(11) $$\begin{array}{l} P=\frac{\left|V_{NR}\cap V_{H}\right|}{\left|V_{NR}\right|} \end{array}$$

*****S*****_*****Global*****_: Combining the comparisons of position of center of mass, image size, image orientation and voxel accuracy, we defined the global similarity between the human- and *NR*-segmented images as:

(12) $$\begin{array}{l} S_{Global}\\ =\frac{\left(1-D_{RG}\right)+\left(1-D_{CM}\right)+\left(1-D_{I}\right)+\left(1-D_{PA}\right)+R}{5} \end{array}$$

*D*_*CM*_, *D*_*RG*_, *D*_*I*_, *D*_*PA*_ and *R* are all between 0 and 1 by definition. The value of *S*_*Global*_ lies between 0 and 1. Note that the precision *P* is not included in the definition of *S*_*Global*_. As described previously, *NR* to segmented more details from the raw image. On the other hand, human tended to segment cleaner and sharper images. The fibers in the *NR* segmented image were usually thicker than the human segmented one. This caused the number of voxels in *NR* segmented image always larger than the human segmented one because of the large surface-volume ratio of the tree-like neuronal structure. Those extra voxels of the real features would be falsely counted in the “false positive” part and lower the precision. As a result, *P* values were not high for those neurons which were classified as “matched” according to the visual validation by biologists (red bars in Figure 4f). On the other hand, some of the broken cases had higher *P* because they didn't have those extra voxels. Thus, *P* was not included in the calculation of *S*_*Global*_. Those real false positive voxels which were not from the reason above would be reflected in the *D* values and decrease the global similarity.

## Data Availability Statement

Publicly available datasets were analyzed in this study. This data can be found here: NeuroRetriever is available on the FlyCircuit webpage http://www.flycircuit.tw/ (Analysis → NeuroRetriever). Quick start guide and tutorial are provided in the webpage.

## Author Contributions

C-TS: original idea for the *NeuroRetriever* workflow, concept, and coding of BRS scoring system. N-YC: concept and coding of *FAST*. T-YW: data management, results validation, and visualization. G-WH: concept and coding of the soma detection. G-TW: *NeuroRetriever* internet interface. Y-JL: data management. T-KL: *FAST* concept. A-SC: HDR concept, supervising acquisition, and management of image data. C-TS, T-KL, and A-SC: supervising the cross-group collaboration and manuscript writing. All authors contributed to the article and approved the submitted version.

## Conflict of Interest

The authors declare that the research was conducted in the absence of any commercial or financial relationships that could be construed as a potential conflict of interest.

## Publisher's Note

All claims expressed in this article are solely those of the authors and do not necessarily represent those of their affiliated organizations, or those of the publisher, the editors and the reviewers. Any product that may be evaluated in this article, or claim that may be made by its manufacturer, is not guaranteed or endorsed by the publisher.

## Supplementary Material

Supplementary Figure 1 Flowcharts of *FAST* processing.

Supplementary Figure 2 Segmentation of a visual neuron (Trh-F-000025) under various intensity thresholds. BRS accumulation (right) solves the segmentation dilemma of skeleton tracing under a fixed threshold (left). Intensity thresholds from **(A–J)** were 2–20, with intervals of two. This is an eight-bit image and the saturating intensity is 255.

Supplementary Figure 3 Pros and cons of *NR* versus human segmentation. **(a)** A putative glutamatergic neuron (VGlut-F-600204) segmented by *NR* (green) showing incomplete fibers as a result of disrupted GFP signals (arrow) that was amended by human segmentation (magenta). **(b)** A putative glutamatergic neuron (VGlut-F-800082) segmented by *NR* (green) showing tangled fibers with a neighboring neuron as a result of oversaturated GFP signals (arrow) that was separated by human segmentation (magenta). **(c)** A raw image with noisy background containing a VGlut-F-200267 olfactory projection neuron (box). Axonal terminals in the calyx was overlooked by human segmentation (magenta) but segmented perfectly by *NR* (green).

Supplementary Figure 4 Global similarity of the matched and unmatched (broken + tangled) cases. Almost all global similarities of the matched cases are larger than 0.7. Global similarity of the unmatched cases distributed broadly from 0 to 1.

Supplementary Figure 5 Examples of high *GS* but unmatched results. In Supplementary Figure 4, there are many cases with *S*_*Global*_ close to 1 but classified as unmatched (tangled or broken) because of our strict standard for matched. **(a–c)** are human segmented, *NR* segmented, and raw images of the neuron TH-F-300027, respectively. **(a,b)** are almost identical (*S*_*Global*_ = 0.974) except a terminal branch is broken (red rectangle), which causes a missing brain region innervated by the neuron. This case was classified as “broken.” **(d–f)** are human segmented, *NR* segmented, and raw images of the neuron Cha-F-800137, respectively. Many neurons were labeled in the raw image **(f)**. *S*_*Global*_ = 0.986 for this case. An extra fiber crosses from a nearby neuron crossed a major fiber and made it classified as “tangled”.

Supplementary Figure 6 New single neuron images found by *NR* from the existing raw images. These neurons were overlooked in the human segmentation process, possibly due to the noisy background or weak signal.

Supplementary Figure 7 Tracing and skeletonization directly from raw brain imaging. Without single-neuron volume segmentation, skeletons reconstructed by the three tracing algorithms, *FAST, APP2*, and *ENT*, are rather inconsistent, even for a single neuron in the brain with clean background. Most importantly, these skeletons do not represent the morphology of the neuron.
