# Supplementary figures and images for: NeuroRetriever: Automatic Neuron Segmentation for Connectome Assembly
Source: Front Syst Neurosci. 2021 Jul 23;15:687182. doi: 10.3389/fnsys.2021.687182 (PMC8342815; doi:10.3389/fnsys.2021.687182)

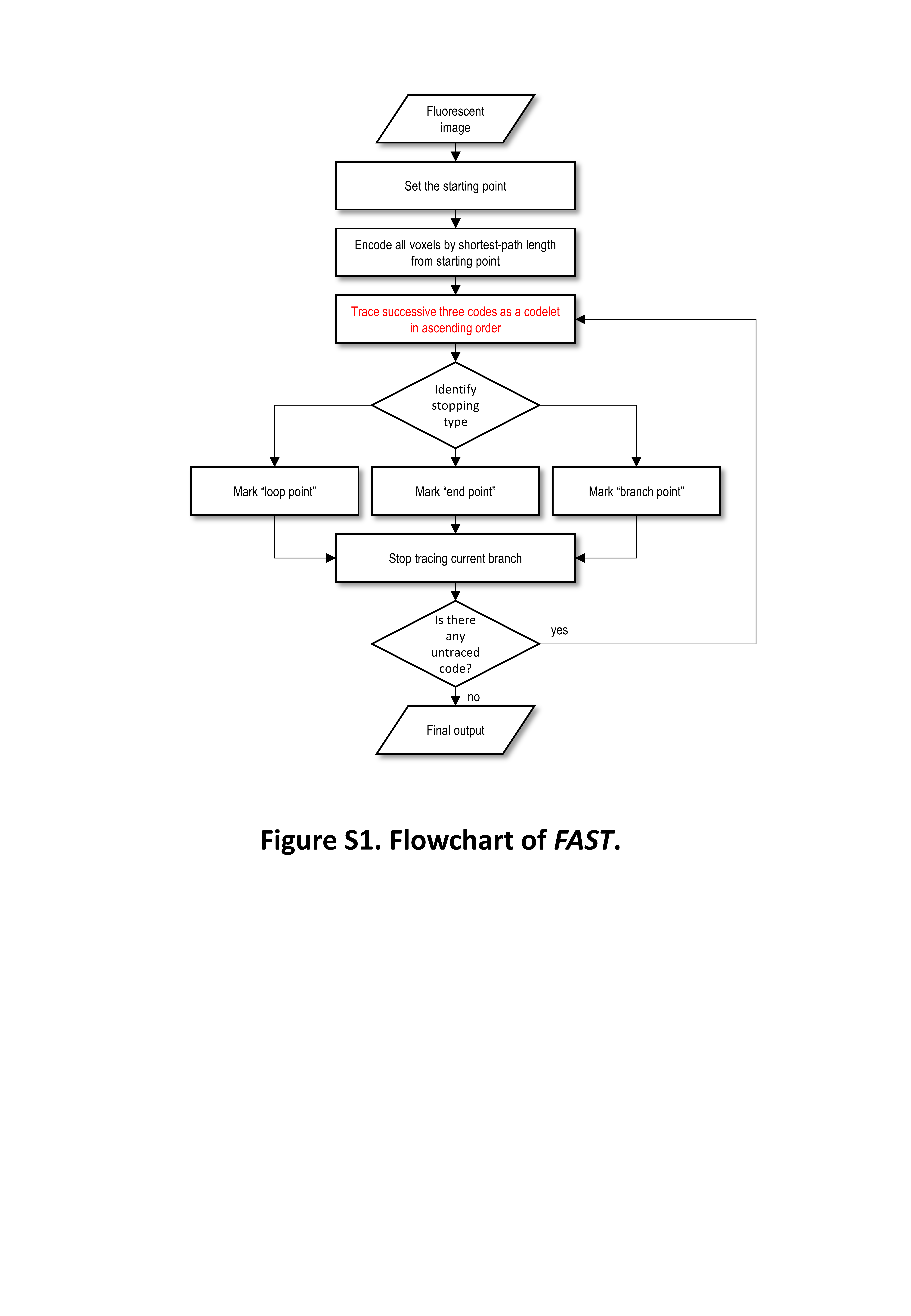

Supplement: Supplementary Figure 1 — Flowcharts of FAST processing. [file Image_1.TIF]

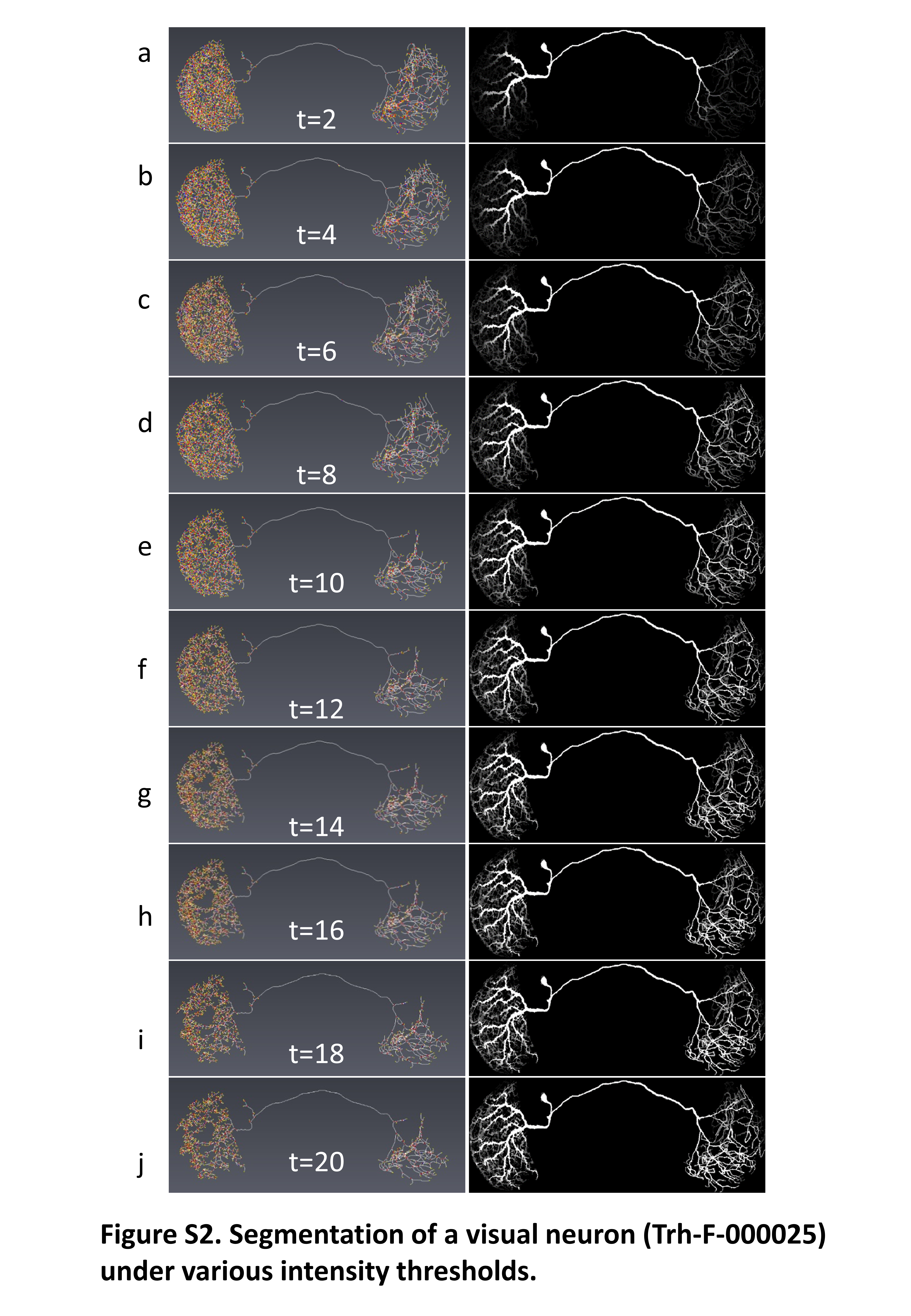

Supplement: Supplementary Figure 2 — Segmentation of a visual neuron (Trh-F-000025) under various intensity thresholds. BRS accumulation (right) solves the segmentation dilemma of skeleton tracing under a fixed threshold (left). Intensity thresholds from (A–J) were 2–20, with intervals of two. This is an eight-bit image and the saturating intensity is 255. [file Image_2.TIF]

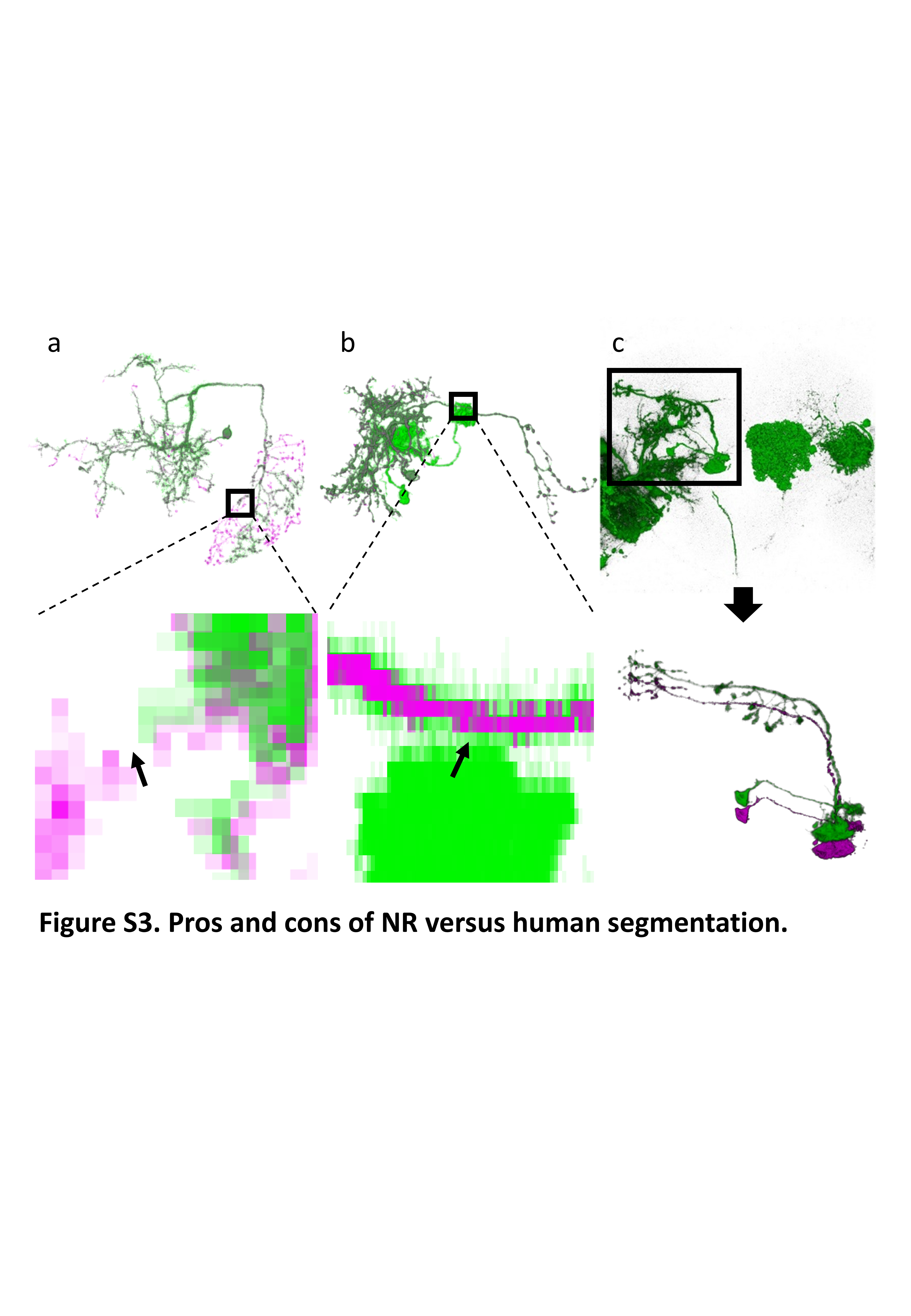

Supplement: Supplementary Figure 3 — Pros and cons of NR versus human segmentation. (a) A putative glutamatergic neuron (VGlut-F-600204) segmented by NR (green) showing incomplete fibers as a result of disrupted GFP signals (arrow) that was amended by human segmentation (magenta). (b) A putative glutamatergic neuron (VGlut-F-800082) segmented by NR (green) showing tangled fibers with a neighboring neuron as a result of oversaturated GFP signals (arrow) that was separated by human segmentation (magenta). (c) A raw image with noisy background containing a VGlut-F-200267 olfactory projection neuron (box). Axonal terminals in the calyx was overlooked by human segmentation (magenta) but segmented perfectly by NR (green). [file Image_3.TIF]

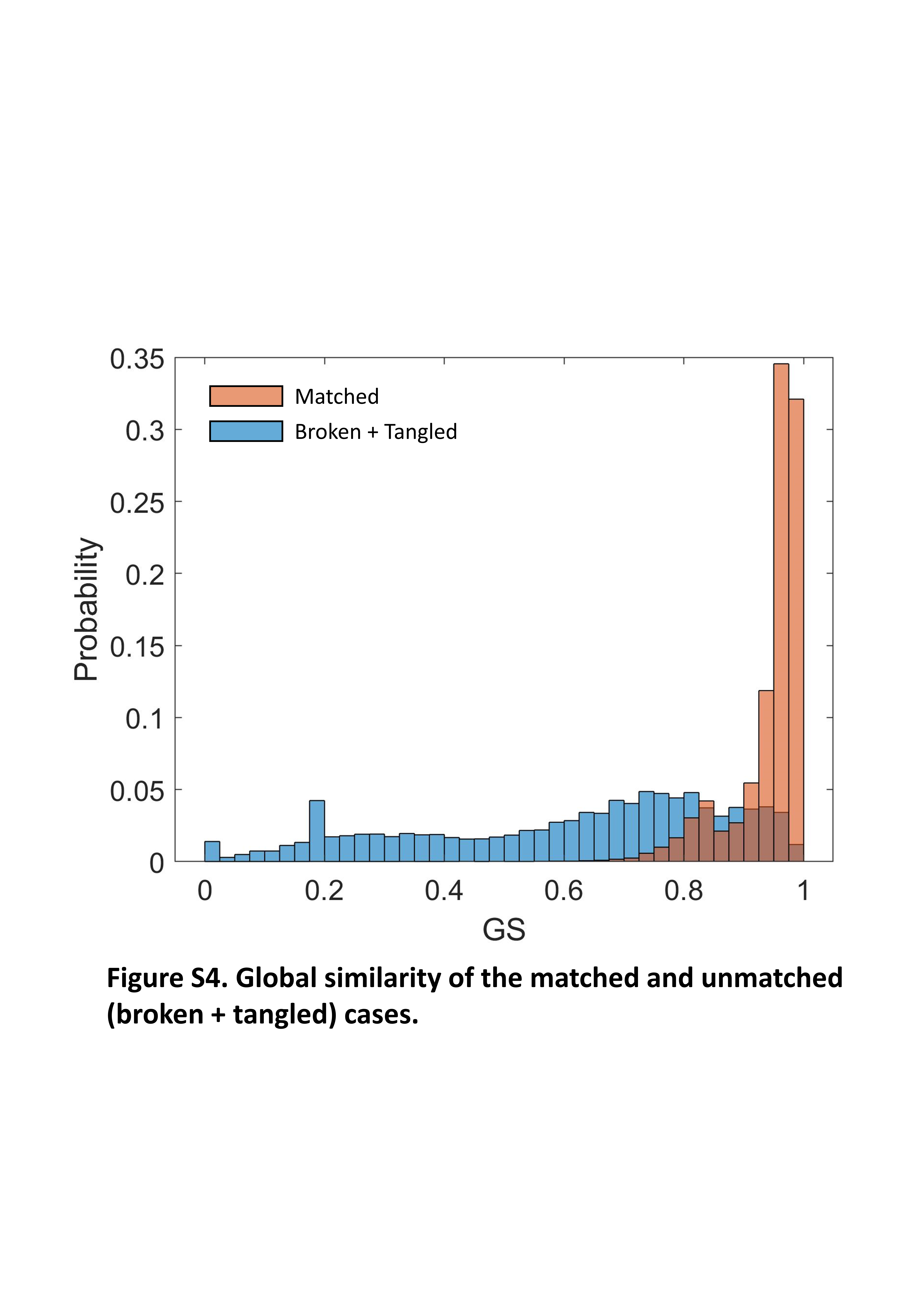

Supplement: Supplementary Figure 4 — Global similarity of the matched and unmatched (broken + tangled) cases. Almost all global similarities of the matched cases are larger than 0.7. Global similarity of the unmatched cases distributed broadly from 0 to 1. [file Image_4.TIF]

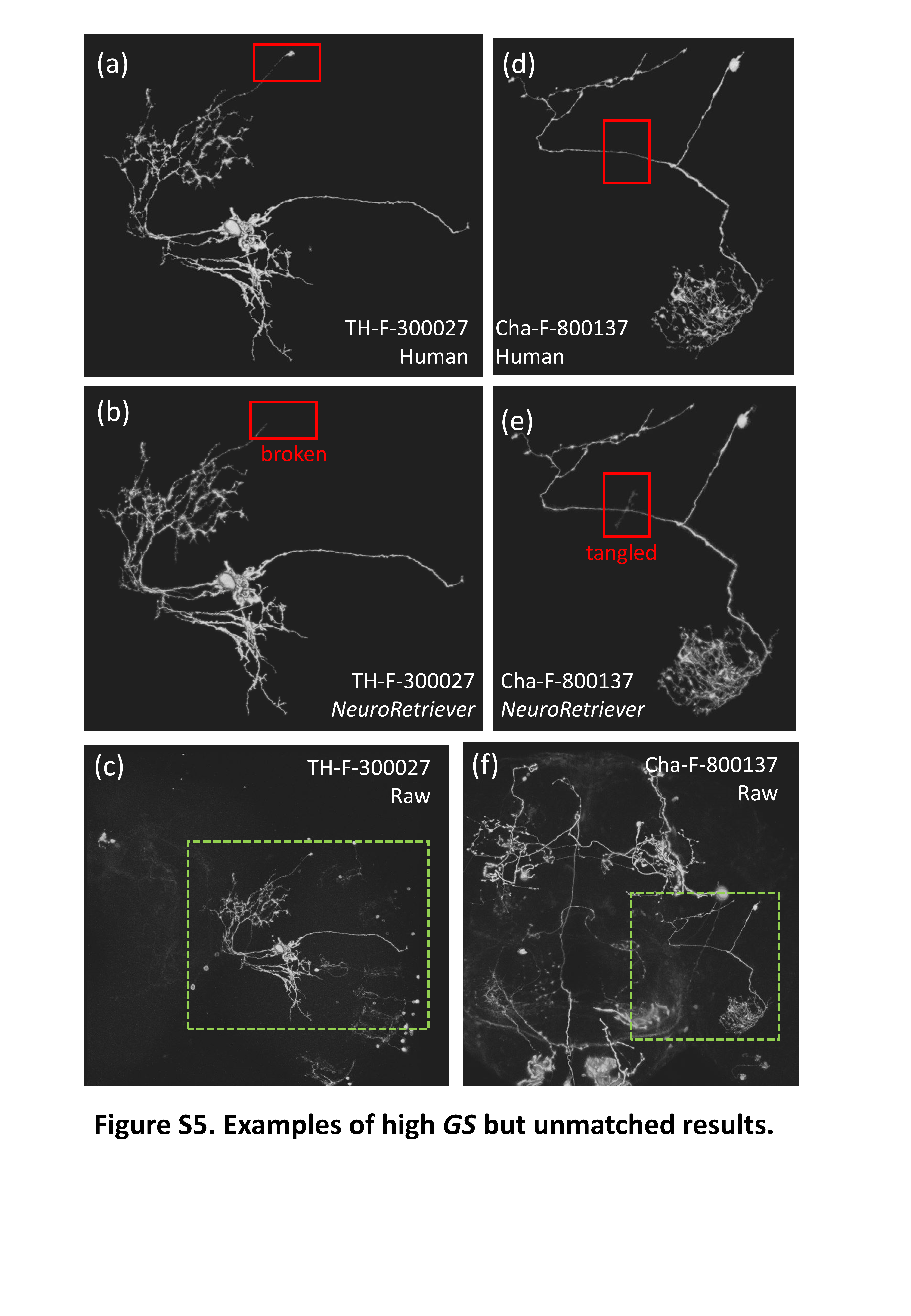

Supplement: Supplementary Figure 5 — Examples of high GS but unmatched results. In Supplementary Figure 4, there are many cases with SGlobal close to 1 but classified as unmatched (tangled or broken) because of our strict standard for matched. (a–c) are human segmented, NR segmented, and raw images of the neuron TH-F-300027, respectively. (a,b) are almost identical (SGlobal = 0.974) except a terminal branch is broken (red rectangle), which causes a missing brain region innervated by the neuron. This case was classified as “broken.” (d–f) are human segmented, NR segmented, and raw images of the neuron Cha-F-800137, respectively. Many neurons were labeled in the raw image (f). SGlobal = 0.986 for this case. An extra fiber crosses from a nearby neuron crossed a major fiber and made it classified as “tangled”. [file Image_5.TIF]

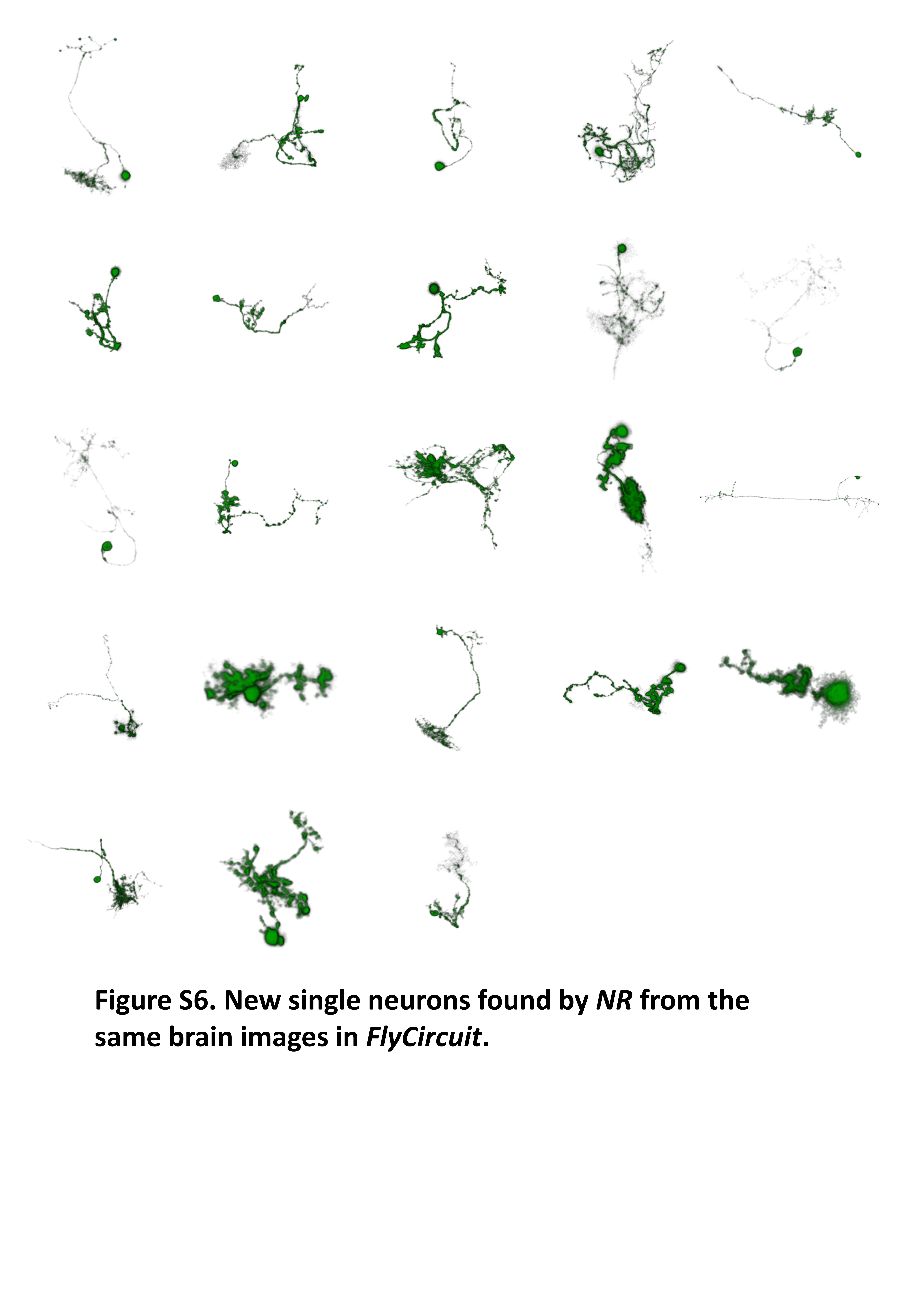

Supplement: Supplementary Figure 6 — New single neuron images found by NR from the existing raw images. These neurons were overlooked in the human segmentation process, possibly due to the noisy background or weak signal. [file Image_6.TIF]

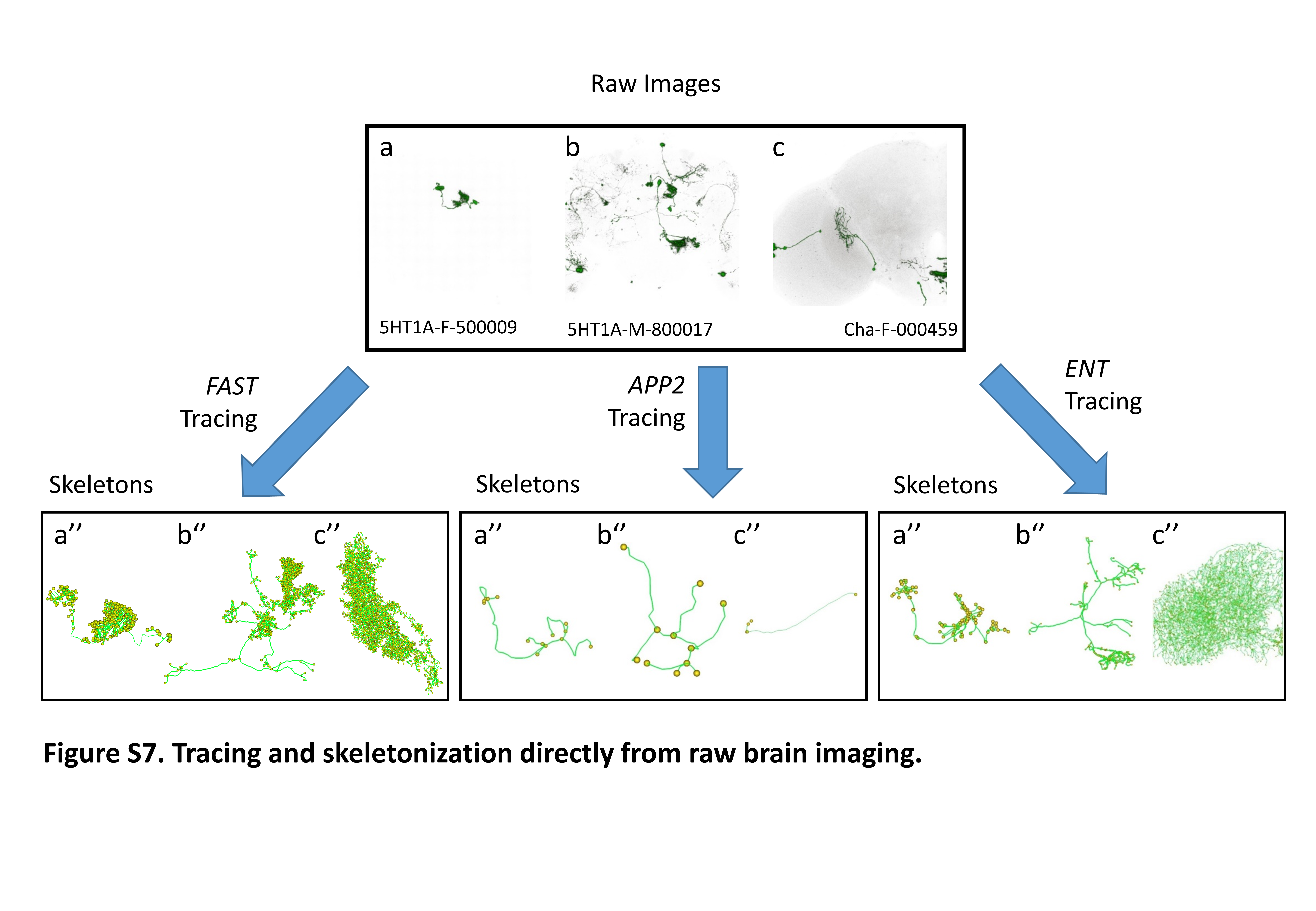

Supplement: Supplementary Figure 7 — Tracing and skeletonization directly from raw brain imaging. Without single-neuron volume segmentation, skeletons reconstructed by the three tracing algorithms, FAST, APP2, and ENT, are rather inconsistent, even for a single neuron in the brain with clean background. Most importantly, these skeletons do not represent the morphology of the neuron. [file Image_7.TIF]
